# Supplementary material for: Hydroxide promotes carbon dioxide electroreduction to ethanol on copper via tuning of adsorbed hydrogen
Source: Nat Commun. 2019 Dec 20;10:5814. doi: 10.1038/s41467-019-13833-8 (PMC6925210; doi:10.1038/s41467-019-13833-8)
Supplement: Supplementary file 1 — Supplementary information [file 41467_2019_13833_MOESM1_ESM.pdf]

Supplementary information for

Hydroxide promotes carbon dioxide electroreduction to ethanol on Cu via tuning of adsorbed hydrogen

Luo *et al.*

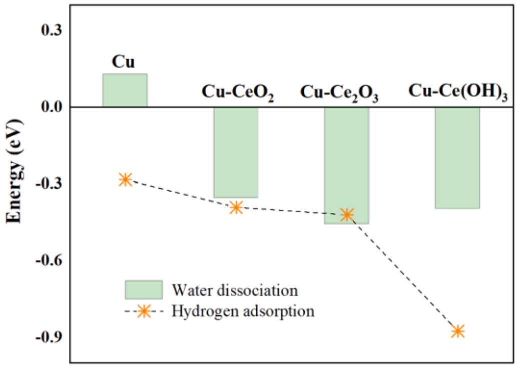

Supplementary Figure 1. Calculated water dissociation reaction energies and hydrogen adsorption energies on bare Cu, CeO_2-doped, Ce_2O_3-doped and $Ce(OH)_3$-doped Cu surfaces.

We carried out DFT calculations to study the role of Ce(III) in the water dissociation and the hydrogen adsorption on Cu, similar to what we did for Ce(IV). As can be seen from Supplementary Figure 1, the water dissociation energy and hydrogen adsorption energy of Ce_2O_3-doped Cu are -0.42 and -0.45 eV, respectively, which are close to those of CeO_2-doped Cu while stronger than that of bare Cu. We also calculated both energies on $Ce(OH)_3$-doped Cu surface, which show water dissociation and hydrogen adsorption energy of -0.39 and -0.87 eV, respectively. Therefore, we believe that all these Ce species have similar enhancement on the ethanol production in CO_2RR by promoting surface H_{ad}.

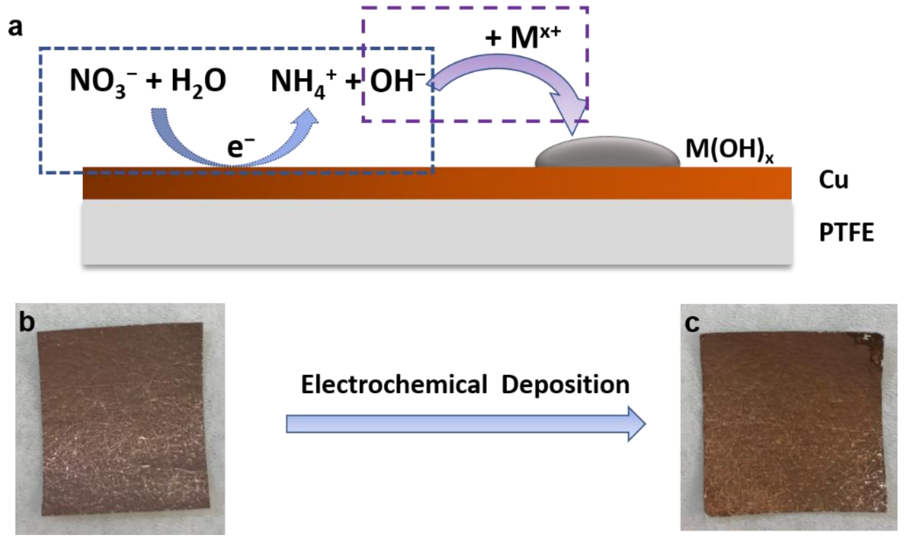

Supplementary Figure 2. Electrochemical deposition of metallic hydroxides. (a) Schematic illustration of electrochemical deposition of metallic hydroxides via the triggering of nitrate reduction. The picture of Cu/PTFE sample before (b) and after (c) electrochemical deposition.

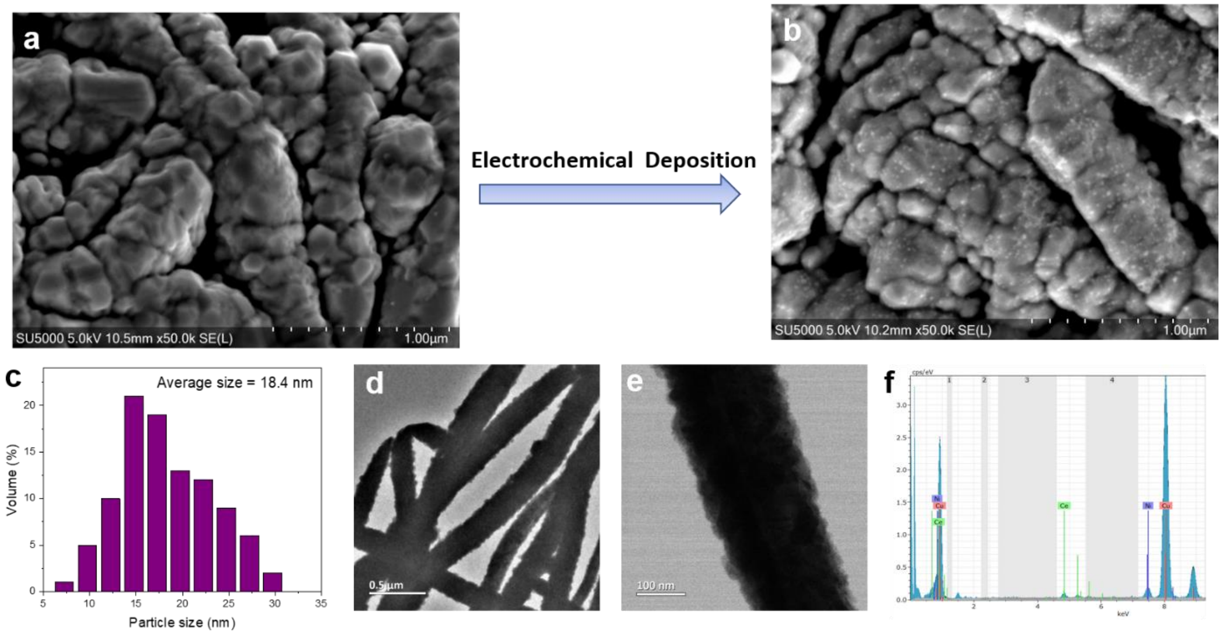

Supplementary Figure 3. Further characterizations of $Ce(OH)_x$/Cu/PTFE. SEM image of Cu/PTFE (a) and $Ce(OH)_x$/Cu/PTFE (b), clearly revealing the emergence of homogeneously dispersed nano-islands on the sputtered Cu surface after electrochemical deposition. (c) The corresponding size-distribution histogram of cerium hydroxide nano-islands on Cu/PTFE, showing a mean size of 18.4 nm. (d, e) TEM images of $Ce(OH)_x$/PTFE, and (f) corresponding TEM-EDX of $Ce(OH)_x$/Cu/PTFE, indicating a co-presence of Cu and Ce.

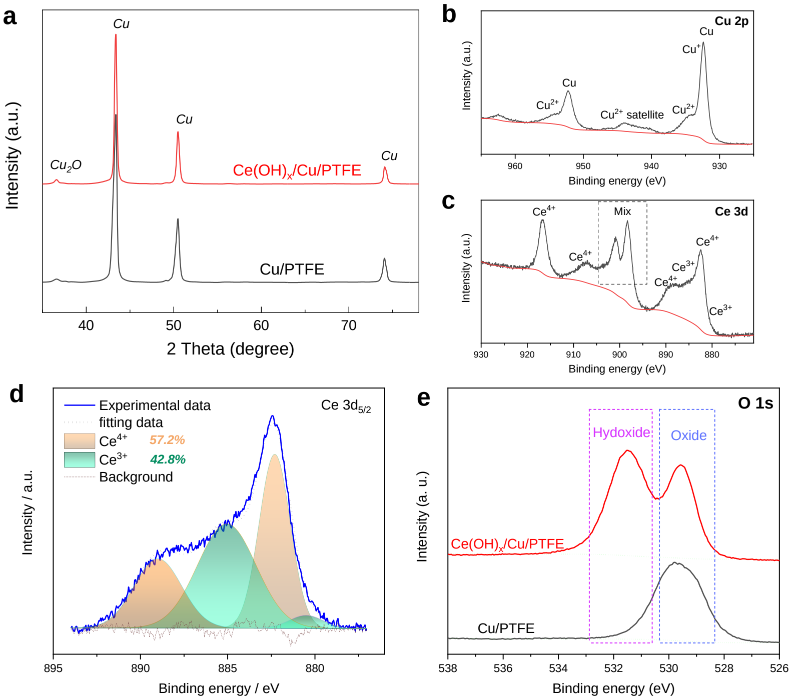

Supplementary Figure 4. (a) x-ray diffraction pattern of $Ce(OH)_x$/Cu/PTFE and bare Cu/PTFE electrode. (b) High-resolution x-ray photoelectron spectroscopy of Cu (2p) and (c) Ce (3d). (d) The experimental and fitting XPS spectra of the Ce $3d_{5/2}$ in $Ce(OH)_x$/Cu/PTFE sample. The ratio of Ce^{4+} and Ce^{3+} was determined to be 57.2% and 42.8%, respectively. (e) High resolution XPS spectra of the O 1s region of Cu/PTFE and $Ce(OH)_x$/Cu/PTFE. The emergence of hydroxide-characteristic oxygen peak of $Ce(OH)_x$/Cu/PTFE indicates the electrochemically-deposited Ce specie exists as hydroxide.

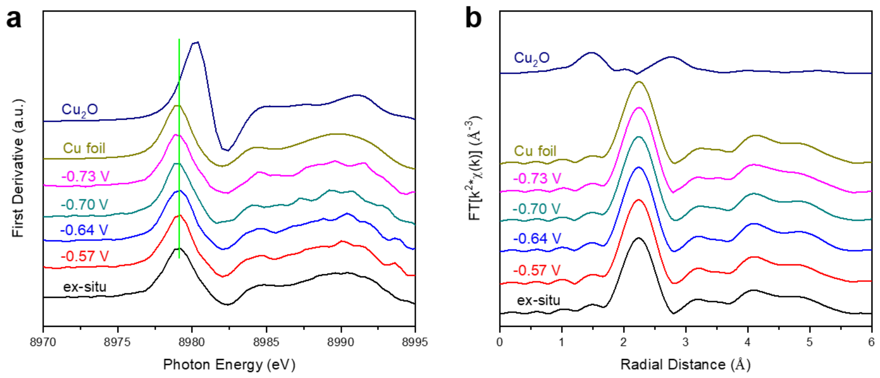

Supplementary Figure 5. (a) Normalised and first derivative Cu K-edge XANES **(b)** Cu K-edge EXAFS spectra of $Ce(OH)_x/Cu/PTFE$.

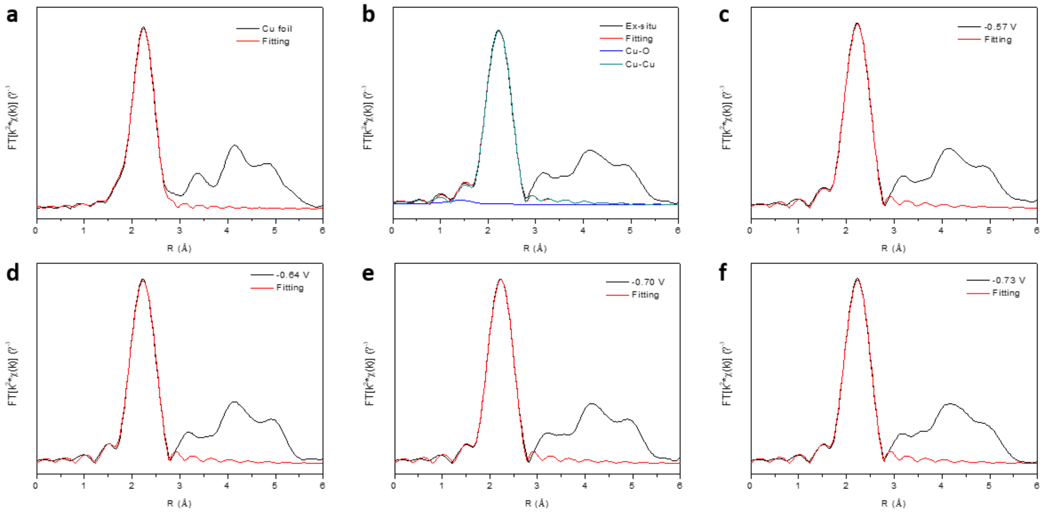

Supplementary Figure 6. Fitting of the Cu K-edge EXAFS spectra recorded at R-space for Cu foil **(a)** and $Ce(OH)_x$/Cu/PTFE catalyst before **(b)** and during **(c-f)** CO_2RR at various potentials.

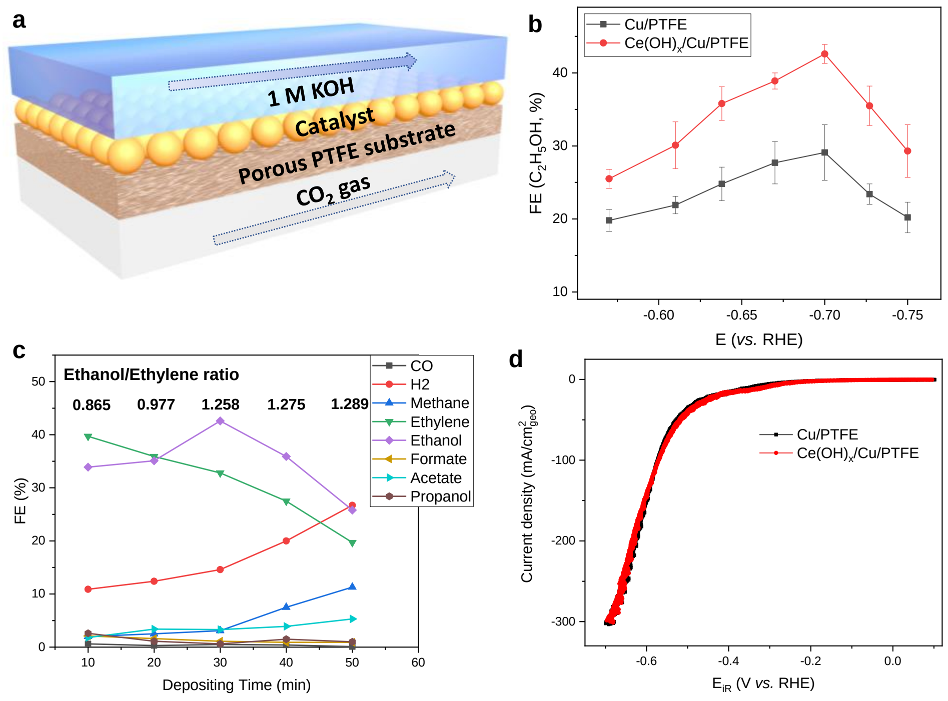

Supplementary Figure 7. (a) Schematic illustration of the employed flow-cell system using Cu/PTFE as the cathode for CO_2RR. (b) Faradaic efficiencies of ethanol on Cu/PTFE and $Ce(OH)_x$/Cu/PTFE under different applied potentials. (c) Product spectrum of $Ce(OH)_x$/Cu/PTFE electrode with different electrochemical depositing time. Increasing the depositing time results in higher surface coverage of $Ce(OH)_x$. (d) CO_2RR polarization curves for Cu/PTFE and $Ce(OH)_x$/Cu/PTFE, recorded under the same conditions as for CO_2RR performance evaluation.

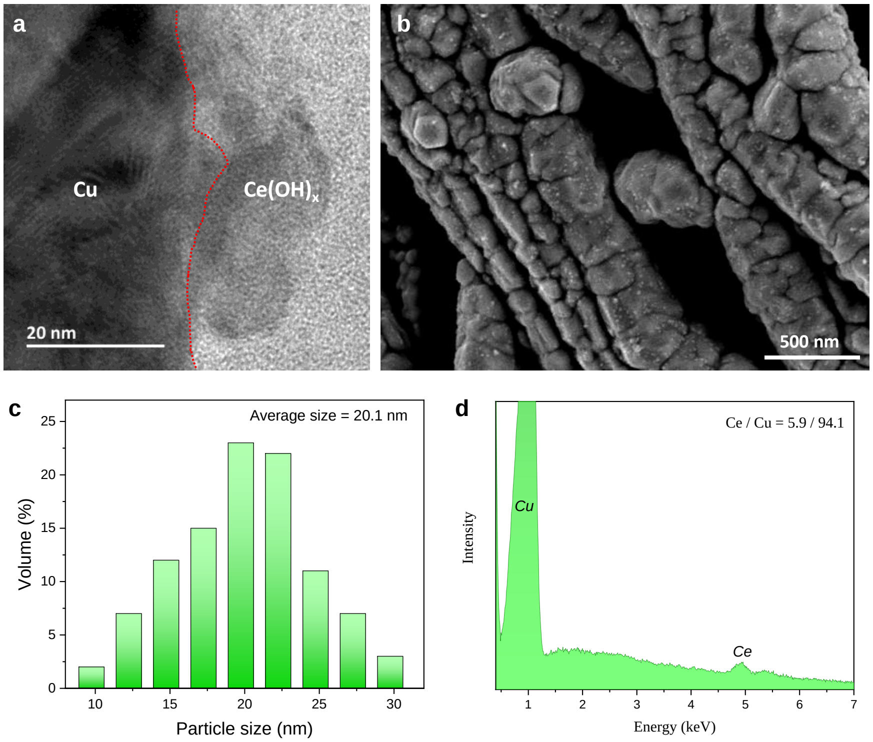

Supplementary Figure 8. (a) TEM image, (b) SEM image, and (c) corresponding size-distribution histogram of cerium hydroxide nano-islands in the sample after CO_2RR process. (d) The EDX spectra of $Ce(OH)_x$/Cu/PTFE electrode after 6-h CO_2RR electrolysis, indicating a Ce/Cu atomic ratio of 5.9/94.1.

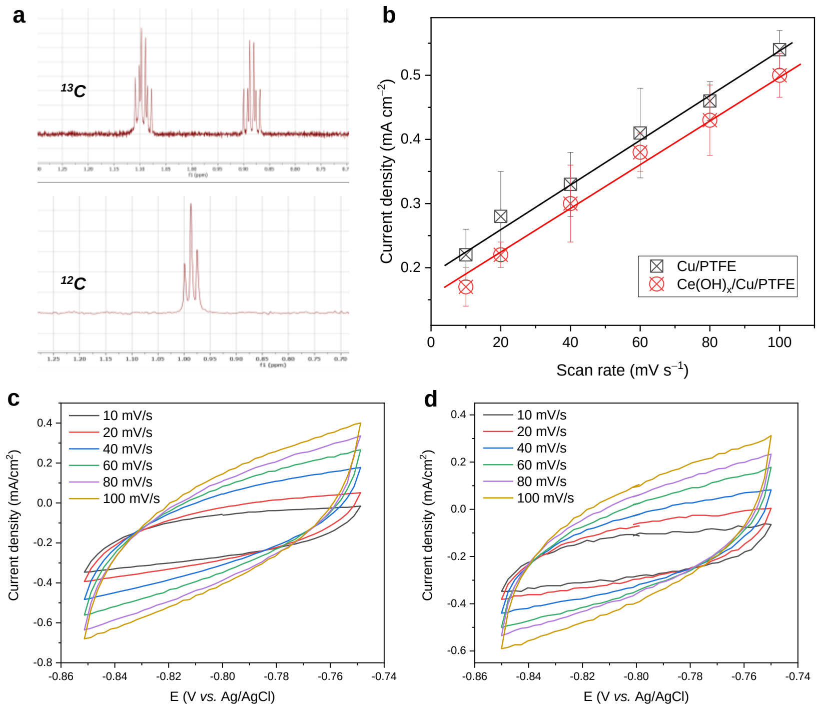

Supplementary Figure 9. (a) ^{1}H-NMR spectrum of ethanol produced from CO_2RR with and without ^{13}C label. (b) Evaluation of the double layer capacitance for Cu/PTFE and $Ce(OH)_x$/Cu/PTFE electrodes. CVs were recorded in the non-Faradaic potential region at scan rates of 10, 20, 40, 60, 80, 100 mV s using flow cell in 1 M KOH in N_2 atmosphere. The capacitance values for Cu/PTFE and $Ce(OH)_x$/Cu/PTFE were determined to be 3.5 and 3.4 mF, respectively. (c) CVs of Cu/PTFE and (b) $Ce(OH)_x$/Cu/PTFE electrodes for double-layer capacitance measurements, recorded in the non-Faradaic potential region at scan rates of 10, 20, 40, 60, 80, 100 mV s^{-1} using flow cell in 1 M KOH in N_2 atmosphere.

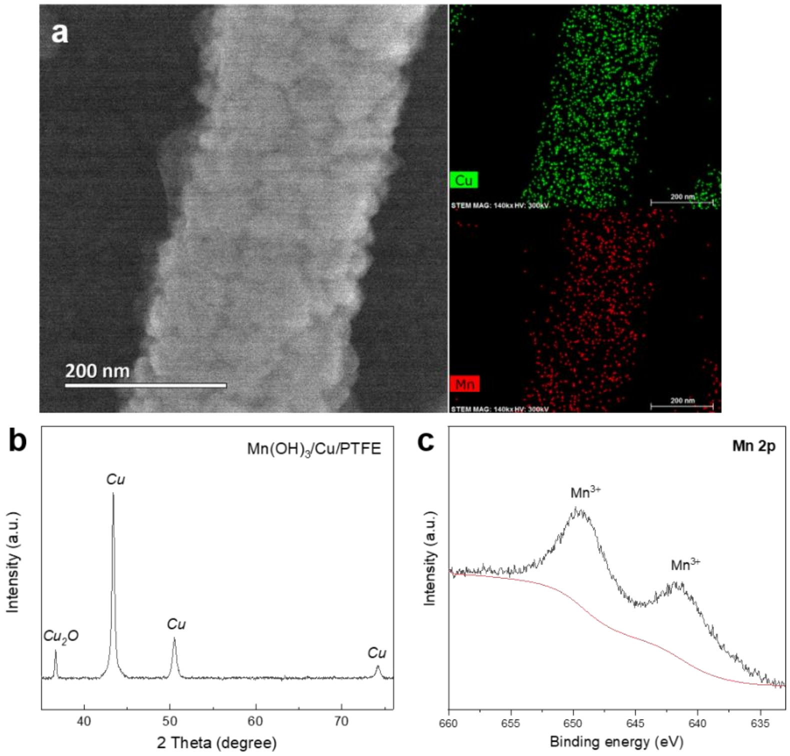

Supplementary Figure 10. Structural characterizations of $Mn(OH)_3$/Cu/PTFE. (a) STEM image and corresponding EDX mapping for Cu and Mn, (b) XRD pattern, and (c) High-resolution XPS of Mn (2p).

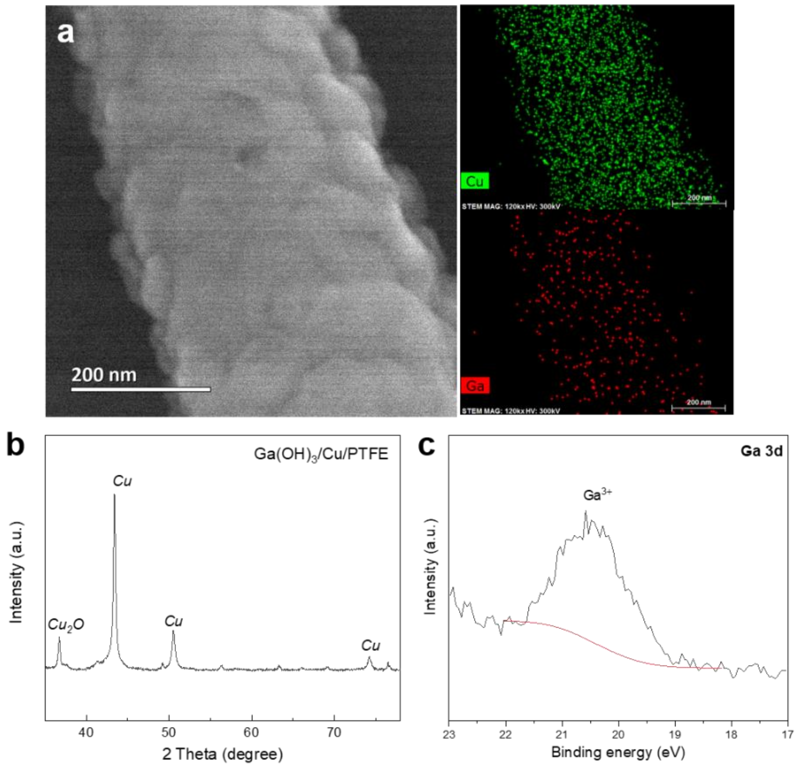

Supplementary Figure 11. Structural characterizations of $Ga(OH)_3$/Cu/PTFE. (a) STEM image and corresponding EDX mapping for Cu and Ga, (b) XRD pattern, and (c) High-resolution XPS of Ga (3d).

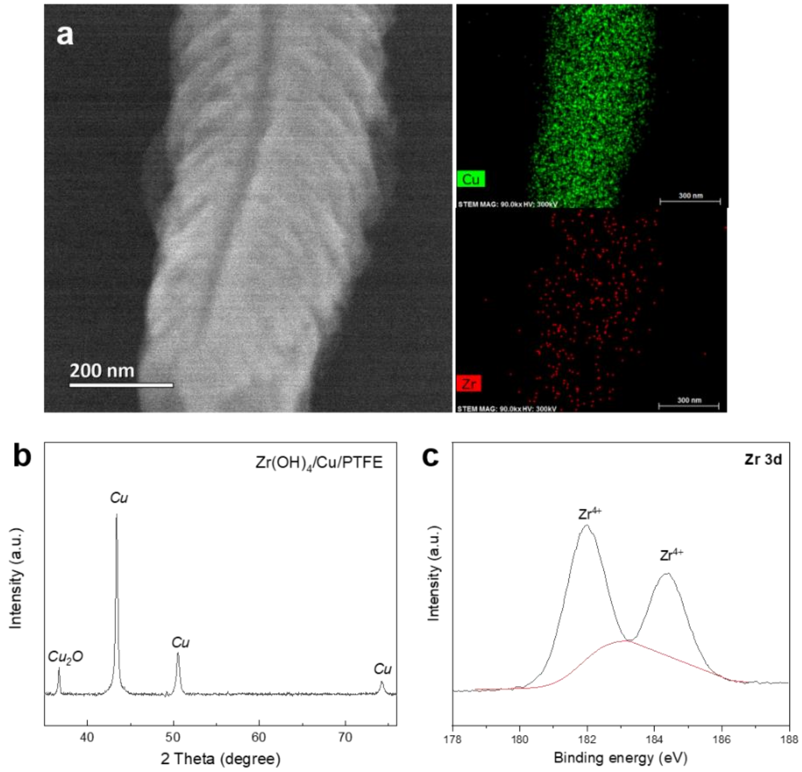

Supplementary Figure 12. Structural characterizations of $Zr(OH)_4$/Cu/PTFE. (a) STEM image and corresponding EDX mapping for Cu and Zr, (b) XRD pattern, and (c) High-resolution XPS of Zr (3d).

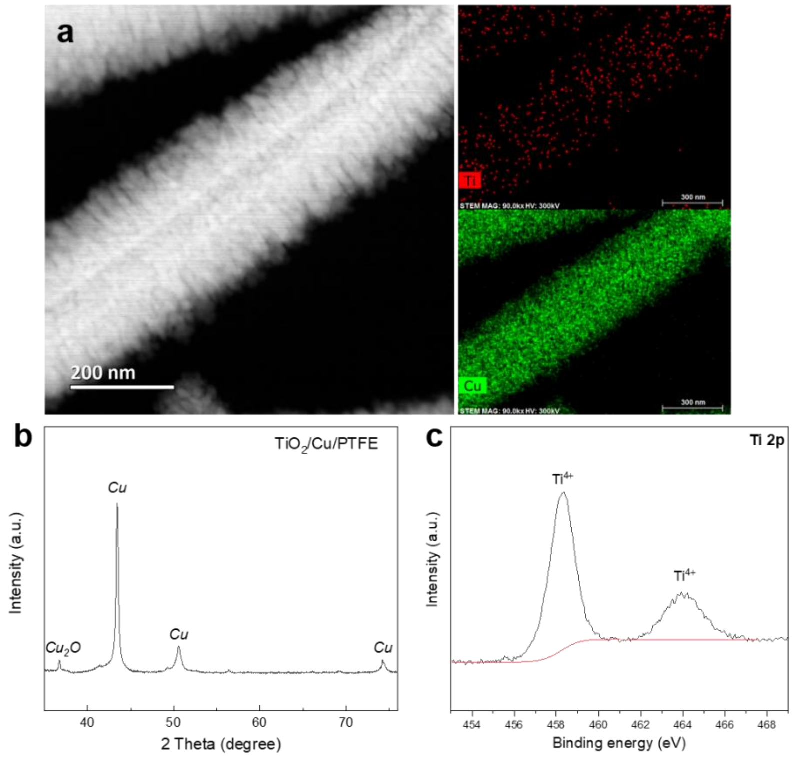

Supplementary Figure 13. Structural characterizations of TiO_2/Cu/PTFE. (a) STEM image and corresponding EDX mapping for Cu and Ti, (b) XRD pattern, and (c) High-resolution XPS of Ti (2p).

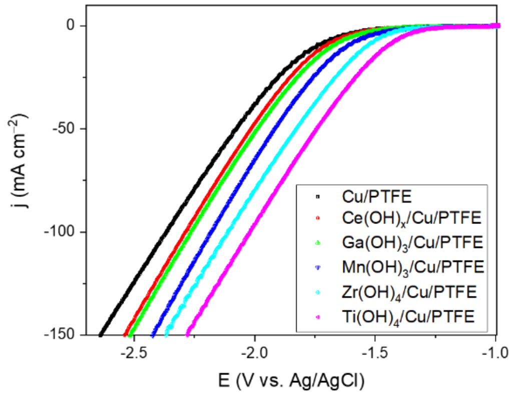

Supplementary Figure 14. HER polarization curves of various metallic hydroxides/oxides modified Cu/PTFE electrodes in Ar-saturated 1 M KOH using flow cell system.

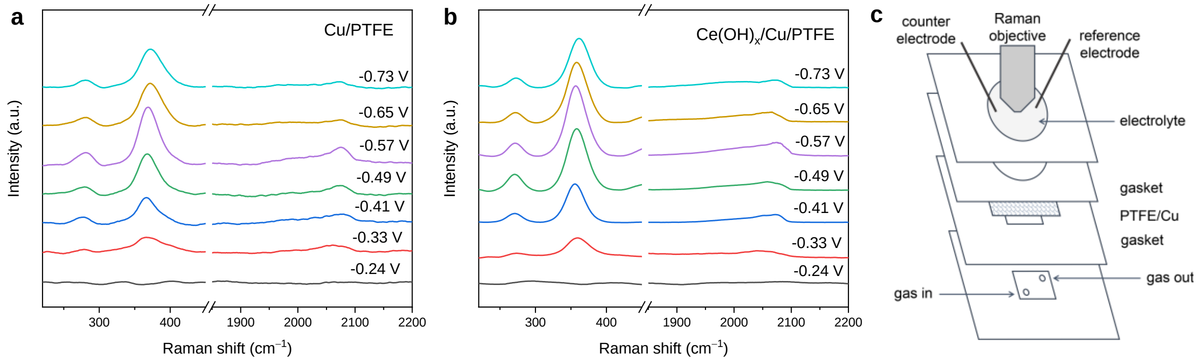

Supplementary Figure 15. (a) Operando Raman spectra of Cu/PTFE and (b) $Ce(OH)_x$/Cu/PTFE electrode in flow cell with 1 M KOH as the electrolyte at various electrochemical potentials, after background subtraction. (c) Schematic illustration of the electrochemical cell for operando Raman measurements. A water immersion objective and a 785 nm laser were used. An Ag/AgCl (3M KCl) electrode and a Pt wire were used as the reference and counter electrodes, respectively.

We conducted in-situ Raman measurements to probe the adsorption behavior of carbon-based intermediates on both bare Cu/PTFE and $Ce(OH)_x$/Cu/PTFE within an operational potential region of −0.24 to −0.73 V in CO_2RR condition. Spectra of two regions are of particular interests to the mechanistic study of CO_2RR, and thus highlighted. Peaks between 250 and 380 cm^{-1} represent the frustrated rotational mode of CO and Cu-CO stretch, while peaks between 1900 and 2150 represent the C≡O stretch of adsorbed CO (CO_{ad}). The comparison of in-situ Raman spectra shows the presence of $Ce(OH)_x$ induce negligible influences on the CO_{ad} behavior of Cu surface. Therefore, the electrocatalytic differences between bare Cu/PTFE and $Ce(OH)_x$/Cu/PTFE mainly derive from the distinctions in H_{ad}.

Supplementary Table 1. Local structure of Cu during CO_2RR process. Fitting of the Cu K-edge EXAFS spectra recorded at R-space for Cu foil (a) and $Ce(OH)_x$/Cu/PTFE catalyst before (b) and during (c-f) CO_2RR at various potentials.

	Scatter	CN	R(Å)	ΔE (eV)	σ^2 (x 10^{-3} Å^2)
Cu foil	Cu-Cu	12	2.54(3)	4.5(5)	8.62
Ex-situ	Cu-O	0.2(4)	1.83(6)	8.1(9)	8.47
	Cu-Cu	11.2(9)	2.54(0)		
-0.57 V	Cu-Cu	11.4(5)	2.53(4)	5.1(0)	8.06
-0.64 V	Cu-Cu	11.3(9)	2.53(4)	5.0(3)	8.03
-0.70 V	Cu-Cu	11.5(7)	2.53(5)	5.2(8)	8.10
-0.73 V	Cu-Cu	11.2(5)	2.53(4)	5.5(6)	7.92

Supplementary Table 2. FEs to various CO_2RR products on Cu/PTFE electrodes as a function of working potential.

E (V *vs.* RHE_{iR})	CO	H_2	CH_4	C_2H_4	Formate	EtOH	Acetate	PrOH
-0.57	6.2 ± 2.7	10.5 ± 1.5	0.4 ± 0.05	41 ± 5	3.3 ± 0.5	19.8 ± 1.5	0.6 ± 0.5	3.7 ± 0.3
-0.61	4.5 ± 1.3	8.8 ± 2	0.7 ± 0.1	44.8 ± 3.1	2.5 ± 0.2	21.9 ± 1.2	1.3 ± 0.2	3.6 ± 0.4
-0.64	3 ± 1.5	6 ± 2.2	1 ± 0.4	52.5 ± 4.5	1.9 ± 0.3	24.8 ± 2.3	2.3 ± 0.5	3.3 ± 0.2
-0.67	2.2 ± 0.8	7.5 ± 1.1	1.3 ± 0.5	48.3 ± 5.5	1.8 ± 0.2	27.7 ± 2.9	2.9 ± 0.4	2.6 ± 0.3
-0.7	1.5 ± 0.5	9 ± 2.5	1.8 ± 0.8	45 ± 3.8	1.3 ± 0.2	29.1 ± 3.8	4.2 ± 0.4	1 ± 0.2
-0.73	1 ± 0.2	11 ± 2.4	3.5 ± 0.6	38.8 ± 4.2	0.8 ± 0.1	23.4 ± 1.4	4.5 ± 0.5	1.2 ± 0.3
-0.75	0.6 ± 0.2	13.4 ± 3.3	7.7 ± 2.9	33.2 ± 3.1	0.5 ± 0.1	20.2 ± 2.1	5.2 ± 0.3	1 ± 0.4

Supplementary Table 3. Current densities of various CO_2RR products on Cu/PTFE electrodes as a function of working potential.

E (V *vs.* RHE_{iR})	CO	H_2	CH_4	C_2H_4	Formate	EtOH	Acetate	PrOH
-0.57	6.2	10.5	0.4	41	3.3	19.8	0.6	3.7
-0.61	6.75	13.2	1.05	66.75	3.75	32.85	1.95	5.4
-0.64	6	12	2	105	3.8	49.6	4.6	6.6
-0.67	5.5	18.75	3.25	120.75	4.5	69.25	7.25	6.5
-0.7	4.5	27	5.6	135	3.9	87.3	12.6	3
-0.73	3.5	38.5	10.5	135.8	2.8	81.9	15.75	4.2
-0.75	2.4	53.6	30.8	132.8	2	80.8	20.8	4

Supplementary Table 4. FEs to various CO_2RR products on $Ce(OH)_x$/Cu/PTFE electrodes as a function of working potential.

E (V *vs.* RHE_{iR})	CO	H_2	CH_4	C_2H_4	Formate	EtOH	Acetate	PrOH
-0.57	3.5 ± 1.9	12.4 ± 2.1	1.2 ± 0.2	31.1 ± 2.1	4.3 ± 1.1	25.5 ± 1.3	0.4 ± 0.1	1.6 ± 0.7
-0.61	2.2 ± 1.3	11 ± 1.8	1.5 ± 0.3	34.5 ± 2.6	2.6 ± 0.9	30.1 ± 3.2	0.5 ± 0.1	1.3 ±0.4
-0.64	1.4 ± 1	10.1 ± 1.1	1.7 ± 0.8	38.7 ± 3.3	1.5 ± 0.3	35.8 ± 2.3	1.1 ± 0.3	0.9 ± 0.2
-0.67	0.8 ± 0.3	12.8 ± 1.7	2.2 ± 1	36.6 ± 3	1.3 ± 0.4	38.9 ± 1.1	1.9 ± 0.4	0.7 ± 0.2
-0.7	0.5 ± 0.25	14.2 ± 2.5	2.9 ± 1.3	33.8 ± 3.4	1.1 ± 0.1	42.6 ± 1.3	3.3 ± 0.5	0.6 ± 0.2
-0.73	0.3 ± 0.1	20.1 ± 3.4	5.5 ± 2.6	28.9 ± 2.2	0.7 ± 0.2	35.5 ± 2.7	5.4 ± 0.8	0.3 ±0.1
-0.75	0.2 ± 0.1	24.8 ± 2.8	9.8 ± 3.2	21. 8 ± 1.2	0.5 ± 0.1	29.3 ± 3.6	7.9 ± 1.2	0.2 ± 0.05

Supplementary Table 5. Current densities of various CO_2RR products on $Ce(OH)_x$/Cu/PTFE electrode as a function of working potential.

E (V *vs.* RHE_{iR})	CO	H_2	CH_4	C_2H_4	Formate	EtOH	Acetate	PrOH
-0.57	3.5	12.4	1.2	31.1	4.3	25.5	0.4	1.6
-0.61	3.3	16.5	2.25	51.75	3.9	45.15	0.75	1.95
-0.64	2.8	20.2	3.4	77.4	3	71.6	2.2	1.8
-0.67	2	32	5.5	91.5	3.25	97.25	4.75	1.75
-0.7	1.5	42.6	8.7	101.4	3.3	127.8	9.9	1.8
-0.73	1.05	70.35	19.25	101.15	2.45	124.25	18.9	1.05
-0.75	0.8	99.2	39.2	87.2	2	117.2	31.6	0.8

Supplementary Table 6. Performance comparison of various catalysts for CO_2 electro-reduction to ethanol with operational current density larger than 10 mA/cm^2. Energy efficiency (EE) is given for the half-cell by assuming no overpotential for the anodic oxygen evolution reaction. Therefore, $EE_{half\ cell}(EtOH) = (1.23 + (-E_{EtOH})) * FE_{EtOH}) / (1.23 + (-E))$, where E_{EtOH} is the thermodynamic potential of CO_2RR to ethanol (+0.09 V versus RHE), E is the applied potential versus RHE and FE_{EtOH} denotes as the Faradaic efficiency for ethanol in percentage.

No.	Catalyst	FE (EtOH, %)	E (vs. RHE)	j (mA/cm^2)	$EE_{half\ cell}$ (EtOH)	J_{EtOH} (mA/cm^2)	EtOH/ethylene ratio	Ref.
1	$Ce(OH)_x$/Cu/PTFE	42.6	-0.7	−300	25.2	127.8	1.26	This work
2	Cu/PTFE	29.1	-0.7	−300	17.2	87.3	0.65	This work
3	Abrupt Cu	11	-0.54	−275	7.1	30.3	0.16	1
4	Cu_2S	25	-0.95	−400	13.7	100	1.16	2
5	Cu(B)	27	-1.1	−70	13.2	18.9	0.52	3
6	CuAg wire	25.9	-0.68	−310	15.4	80.3	0.47	4
7	CuZn	29.1	-1.05	−20	14.5	5.8	2.71	5
8	Nanoporous Cu	16.6	-0.67	−653	9.96	108.4	0.43	6
9	Cu-DAT wire	27.3	-0.69	−170	16.2	46.4	0.77	7
10	Cu_3N	18.4	-0.95	−25	9.6	4.6	0.47	8
11	Cu nanoparticles-4	17.4	-0.79	−300	9.82	52.5	0.38	9

Supplementary references

1 Dinh, C. T. *et al.* CO2 electroreduction to ethylene via hydroxide-mediated copper catalysis at an abrupt interface. *Science* **360**, 783-787 (2018).

2 Zhuang, T.-T. *et al.* Steering post-C–C coupling selectivity enables high efficiency electroreduction of carbon dioxide to multi-carbon alcohols. *Nat. Catal.* **1**, 421-428 (2018).

3 Zhou, Y. *et al.* Dopant-induced electron localization drives CO2 reduction to C2 hydrocarbons. *Nature chemistry*, **10**, 974-980 (2018).

4 Hoang, T. T. H. *et al.* Nanoporous Copper-Silver Alloys by Additive-Controlled Electrodeposition for the Selective Electroreduction of CO2 to Ethylene and Ethanol. *J. Am. Chem. Soc.* **140**, 5791-5797 (2018).

5 Ren, D., Ang, B. S.-H. & Yeo, B. S. Tuning the Selectivity of Carbon Dioxide Electroreduction toward Ethanol on Oxide-Derived CuxZn Catalysts. *ACS Catal.* **6**, 8239-8247 (2016).

6 Lv, J. J. *et al.* A Highly Porous Copper Electrocatalyst for Carbon Dioxide Reduction. *Adv. Mater.* **30**, 1803111-1803119 (2018).

7 Hoang, T. T. H., Ma, S., Gold, J. I., Kenis, P. J. A. & Gewirth, A. A. Nanoporous Copper Films by Additive-Controlled Electrodeposition: CO2 Reduction Catalysis. *ACS Catal.* **7**, 3313-3321 (2017).

8 Liang, Z. Q. *et al.* Copper-on-nitride enhances the stable electrosynthesis of multi-carbon products from CO2. *Nat. Commun.* **9**, 3828- 3836 (2018).

9 Ma, S. *et al.* One-step electrosynthesis of ethylene and ethanol from CO2 in an alkaline electrolyzer. *J. Power Sources* **301**, 219-228 (2016).
